# Lurasidone Augmentation of Clozapine in Schizophrenia—Retrospective Chart Review

**DOI:** 10.3390/brainsci13030445

**Published:** 2023-03-04

**Authors:** Marcin Siwek, Adrian Andrzej Chrobak, Aleksandra Gorostowicz, Patrycja Król, Dominika Dudek

**Affiliations:** 1 Department of Affective Disorders, Jagiellonian University Medical College, Kopernika St. 21a, 31-501 Cracow, Poland; 2 Department of Adult Psychiatry, Jagiellonian University Medical College, Kopernika St. 21a, 31-501 Cracow, Poland; 3 Department of Adult, Child and Adolescent Psychiatry, University Hospital in Cracow, Kopernika St. 21a, 31-501 Cracow, Poland

**Keywords:** clozapine resistance, treatment resistance, augmentation, schizophrenia

## Abstract

The aim of our study was to evaluate the effectiveness of lurasidone augmentation of clozapine in treatment-resistant schizophrenia (SZ) in a retrospective chart review. From the medical records of 916 SZ patients, we identified 16 individuals treated with a combination of clozapine and lurasidone. The detailed clinical data are described separately for each patient. We compared the Clinical Global Impression—Severity (CGI-S) scores between three points of observation: before the treatment and one month and two months after its initiation. CGI Improvement (CGI-I) scores were used to evaluate the treatment response between the first and last points of observation. The vast majority of patients (14/16, 87.5%) responded to lurasidone augmentation of clozapine (CGI-I scores 1 or 2). Therapeutic effects were observable after 3–12 weeks of treatment (median 6 (4–6)). A reduction in CGI-S scores was observed after the first month of observation. There was an observable reduction in positive, depressive and anxiety symptoms, as well as an improvement in psychosocial functioning. Two patients discontinued treatment due to side effects. Our study suggests that lurasidone augmentation of clozapine may lead to improvements in a broad range of SZ symptom dimensions.

## 1. Introduction

Schizophrenia (SZ) is a chronic mental disorder presenting with a variety of symptoms (positive, negative and cognitive) that can lead to the significant deterioration of psychosocial functioning [1]. About 30–50% of SZ patients present treatment resistance, defined as the occurrence of difficult-to-treat psychotic symptoms after at least two subsequential trials of typical or atypical antipsychotics in monotherapy, with the use of a sufficient dose, duration and adherence [2,3]. The only antipsychotic drug recommended by the FDA for treatment-resistant SZ is clozapine, which consequently has been shown to be superior to all other neuroleptics for this condition [4,5,6]. However, the efficacy of clozapine monotherapy is far from optimal, as 40% to 70% of patients fail to respond or present only a partial response to an adequate treatment with the use of this drug [7,8,9,10]. While combining clozapine with other antipsychotics is commonly used in practice in order to overcome the suboptimal effects of clozapine monotherapy, supporting data on the efficacy of such combinations are based on low-quality studies (open-label studies or using observed case data) [11]. In the latest meta-analysis of RCTs (randomized controlled trials) examining treatment options for clozapine-resistant SZ, none of the assessed antipsychotics (quetiapine, pimozide, risperidone, sertindole, ziprasidone, aripiprazole, sulpiride, amisulpride) was observed to be superior to placebo [12]. Moreover, data evaluating the use of the new antipsychotics for this purpose, such as cariprazine and lurasidone, are limited to case reports [13,14,15,16].

Lurasidone is a new-generation antipsychotic drug characterized by a high affinity for dopamine (D_2_) and serotonin 5-HT_2A_ and 5-HT_7_ receptors and a moderate affinity for 5-HT_1A_ and α_2c_ receptors [17]. Contrary to many second-generation antipsychotics, this drug presents either no or minimal affinity for H_1_, M_1_ and 5-HT_2C_ receptors, which are associated with sedation, increased weight and metabolic syndrome [18]. Multiple RCTs proved the efficacy of this drug in SZ patients [19,20,21,22]. A pooled analysis of clinical studies demonstrated that lurasidone treatment improves the full spectrum of SZ symptoms representing all five factors of the Positive and Negative Syndrome Scale (PANSS): positive and negative symptoms, disorganization of thought, hostility/excitement and depression/anxiety [23]. Moreover, it has been shown that this drug reveals promising beneficial effects on cognitive impairments in SZ patients [24].

While there are many studies on the efficacy of the combination of clozapine with other antipsychotic medications [25], to our best knowledge, there is only one report evaluating the results of treatment with lurasidone and clozapine in treatment-resistant SZ [16]. Arienti et al. (2021) presented two cases of SZ patients in whom this combination was used to treat a relapse of psychosis. The patients presented a meaningful decrease in the severity of positive and negative symptoms. Lurasidone augmentation of clozapine showed a good tolerability profile. No major alterations were detected in blood tests or QTc, and no extrapyramidal symptoms were observed during psychiatric hospitalization [16].

Due to the premises of the beneficial effect of the combination of these drugs, the purpose of our study was to assess the effectiveness of lurasidone augmentation of clozapine in treatment-resistant SZ by performing a retrospective chart review.

## 2. Materials and Methods

**Figure 1:**
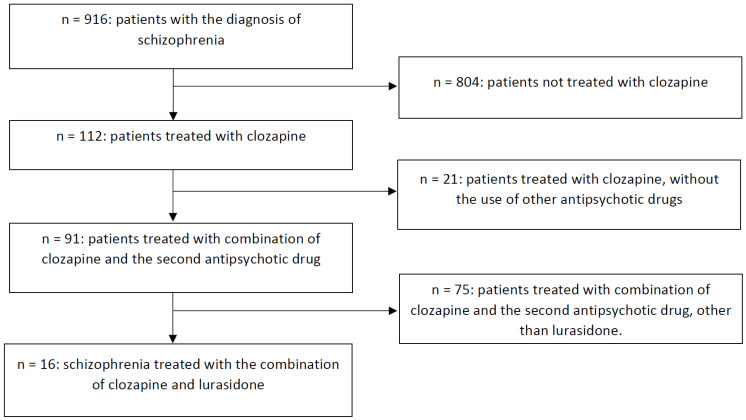
Flow chart of retrospective chart review.

A retrospective chart review was performed to evaluate the effects of lurasidone augmentation of clozapine in patients with difficult-to-treat SZ, according to the methodology of our previous study [26]. A convenience sample strategy was applied as the sampling method. All authors performed the data analysis. The dataset covered the medical records (electronic and paper) of all patients diagnosed with SZ (in accordance with the ICD-10 criteria) treated in the Department of Adult Psychiatry of University Hospital in Cracow between 2018 and 2022. Patients’ data were chosen for the analysis if they met the inclusion criteria: (1) diagnosis of schizophrenia; (2) age > 18 years old; (3) both sexes; (4) treatment with a combination of clozapine and lurasidone. The exclusion criteria were (1) no follow-up observations documented; (2) missing data relevant to the analysis. Figure 1 shows a flow chart of the retrospective chart review. At the beginning of data abstraction, the researchers were trained in order to establish a common procedure for the data analysis. Regular meetings between the researchers were organized in order to discuss potential doubts occurring during the abstraction process. In the case of missing or incomplete data, we contacted the clinician who was taking care of the particular patient. Data-collecting forms have been attached to this article (Annex 1). The medical records of 916 patients diagnosed with SZ were analyzed. In this group, 112 patients were treated with clozapine, of whom 91 were additionally receiving a second antipsychotic drug. In this group, we identified 16 SZ patients receiving treatment with a clozapine and lurasidone combination. The inter-rater reliability was high: there were only several features on which the researchers performing data abstraction disagreed, which gives Cohen’s kappa > 0.8 for all variables. The disagreements were resolved by discussion with other researchers. The intra-rater reliability was very high too and was assessed on a sample of 10 patients, for which Cohen’s kappa was also excellent, with values > 0.8.

In the next step, data were extracted from the SZ patients’ medical records through the use of chart files in the form of an electronic table with the following data: age, sex, duration of SZ treatment, number of previous ineffective pharmacotherapy trials before introducing lurasidone augmentation of clozapine, antipsychotic drug used in combination with clozapine before lurasidone, clozapine dose, somatic comorbidities, substance abuse, psychiatric and nonpsychiatric drugs (and their doses) concomitantly used by the patients, initial and final lurasidone doses, duration of lurasidone augmentation of clozapine treatment, and observable effects of the use of this combination. We evaluated the data for the presence of the following symptoms and conditions: residual positive symptoms, exacerbation of positive symptoms, negative symptoms, anxiety symptoms, suicidal ideations, cognitive impairment, sexual dysfunctions, hyperprolactinemia, increased appetite and weight/obesity, and glucose intolerance (features such as metabolic disorders, hyperprolactinemia, and cognitive dysfunctions were selected due to the reported favorable profile of the lurasidone effect on metabolic parameters and cognitive functions and a possible risk of hyperprolactinemia [27,28]). Moreover, we retrospectively assessed the Clinical Global Impression—Severity (CGI-S) scores before treatment initiation, at months 1, 2, and 3 and then every three months. We evaluated the difference in CGI-S scores within the shortest follow-up period common to all patients (2 months). Thus, mean CGI-S scores representing three observation points (before treatment initiation and one month and two months after treatment initiation) were compared with the use of the repeated-measures ANOVA test. We also analyzed differences in CGI-S scores across the first 6 months of observation. Additionally, we calculated Clinical Global Impression—Improvement (CGI-I) scores in order to assess the treatment response between the first and last points of observation. We classified the patients as responding to lurasidone augmentation of clozapine if they were given 1 or 2 points on the CGI-I scale (“Very much improved” or “Much improved”) at any point of the follow-up. For each of the patients fulfilling the abovementioned criteria, we present the number of weeks of treatment after which therapeutic effects were observable.

## 3. Results

Detailed data extracted from the medical records of 16 patients are presented in Table 1 and summarized in Table 2. Patients’ ages ranged from 27 to 45 years (median 37.5 (28.25–41.5)). There were seven women and nine men. The median duration of the overall SZ treatment was 12.5 years (7–16). All patients presented resistance to antipsychotic treatment. The median number of previous ineffective pharmacotherapy trials prior to the use of the clozapine and lurasidone combination was 5 (3.6–6.75). The characteristics of the psychopathological symptoms and somatic conditions presented by the patients before the initiation of lurasidone augmentation of clozapine are presented in Table 3.

Prior to the switch to lurasidone, 12 patients had had a trial of clozapine augmentation with another antipsychotic: amisulpride (five patients), aripiprazole (four patients), cariprazine (one patient), haloperidol (one patient), and risperidone (one patient). Some patients had had several trials of augmentation of clozapine with other antipsychotics prior to introducing lurasidone: two patients had two previous trials, three had three trials, three had four trials, one had five trials, two had six trials and one had seven trials. In two cases, lurasidone was initially used in combination with olanzapine, with a subsequent switch from olanzapine to clozapine. In one case, the patient used aripiprazole alongside olanzapine prior to the switch to the lurasidone and clozapine combination. The mean daily dose of clozapine combined with lurasidone was 326.6 ± 147.3 mg (dose range 50–500 mg). In every case, the initial lurasidone dose was 37 mg. The final dose ranged from 37 to 148 mg (mean 104.1 ± 41 mg). The duration of the combined treatment lasted between 2 and 38 months (median 8.5 (4–14)).

**Figure 2:**
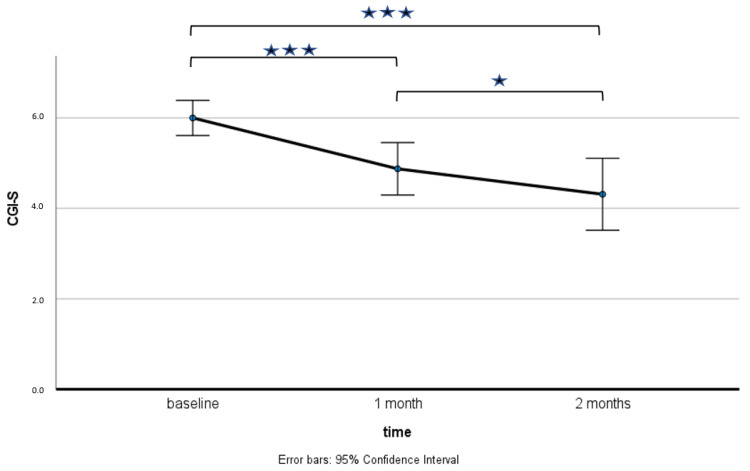
Comparison of Clinical Global Impression—Severity (CGI-S) between three points of observation: before the treatment and one month and two months after the initiation of lurasidone augmentation of clozapine in a group of 16 patients with schizophrenia. * *p* ≤ 0.05, *** *p* ≤ 0.001.

We evaluated the difference in CGI-S scores within the shortest follow-up period common to all patients (2 months). We compared the global severity of patients’ symptoms between the three points of observation: before the treatment and one month and two months after its initiation. The data met the assumption of the repeated-measures ANOVA test, as the residuals follow a normal distribution, which was assessed with the Shapiro–Wilk Test (*p* = 0.425), and Mauchly’s test showed that the assumption of sphericity was not violated (χ2(2) = 3.292, *p* = 0.193). The repeated-measures ANOVA test indicated that there is a significant difference in the CGI-S scores between the three measurement points (F(2, 30) = 36.5, *p* < 0.001), with a mean of 6 ± 0.73 before the treatment, 4.88 ± 1.09 after the first month and 4.31 ± 1.49 after the second month of the trial. The partial effect size ηp2 is 0.7087 (treatment effect size). The post hoc paired *t*-test using a Bonferroni-corrected α = 0.017 indicated significant differences in CGI-S scores between the abovementioned points of observation, as presented in Figure 2.

**Figure 3:**
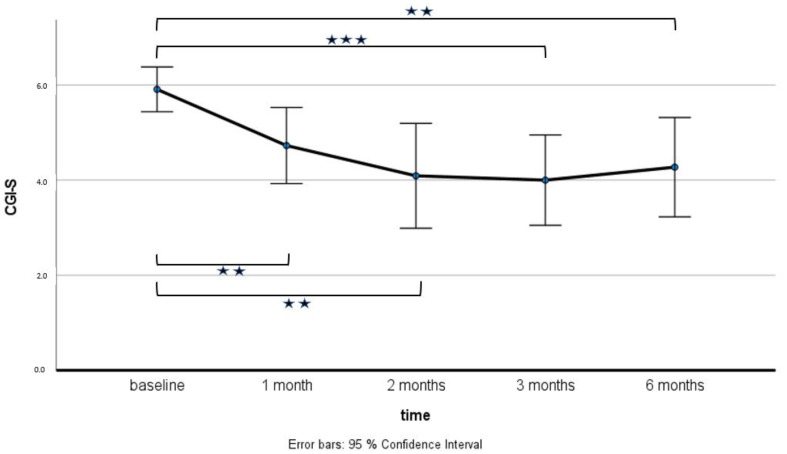
Comparison of Clinical Global Impression—Severity (CGI-S) between five points of observation: before the treatment, one month, two months, three months and six months after the initiation of lurasidone augmentation of clozapine in a group of 11 patients with schizophrenia. ** *p* ≤ 0.01; *** *p* ≤ 0.001.

We also assessed differences in CGI-S scores across the first 6 months of observation. Five points of measurement were included: baseline and after 1, 2, 3 and 6 months. The assumptions of repeated-measures ANOVA were met: the residuals followed a normal distribution (assessed by the analysis of z-scores for skewness and kurtosis: all absolute values were <1.96), and Mauchly’s test indicated a lack of violation of the assumption of sphericity (χ2(9) = 8.798, *p* = 0.465). The repeated-measures ANOVA test demonstrated significant differences in the CGI-S scores across the five time points (F(4, 40) = 18.5, *p* < 0.001), with a mean and standard deviation of 6 ± 0.73 at baseline, 4.88 ± 1.09 after the first month, 4.31 ± 1.49 after two months, 4 ± 1.35 after three months and 4.27 ± 1.56 after six months. The effect size measured by the partial effect size ηp2 was 0.649. The post hoc tests with Bonferroni correction showed statistically significant differences in CGI-S scores between baseline and all follow-up time points, as presented in Figure 3.

Therapeutic effects were observable after 3–12 weeks of treatment (median 6 (4–6)). Fourteen patients (87.5%) showed a response to the treatment (CGI-I scores of 1 or 2, and a CGI-S reduction of 2 points). Two patients (12.5%) showed minimal global improvement. Data extracted from medical records indicated that patients presented an observable reduction in positive symptoms (10 patients), anxiety (7 patients), affective symptoms (7 patients) and sexual dysfunctions (2 patients in whom lurasidone was introduced after discontinuation of risperidone and amisulpride). Improvements in psychosocial functioning were observed in nine individuals, and four patients returned to work. A positive effect on weight (stabilization or its reduction) was shown in two patients in whom lurasidone replaced haloperidol and risperidone. Two patients achieved a normalization of the prolactin level (after switching from risperidone and amisulpride to lurasidone), and in the case of one patient, there was an observable stabilization of glucose levels (after risperidone was replaced by lurasidone). In three patients, the treatment was discontinued: two patients due to the side effects (hyperprolactinemia with galactorrhea or extrapyramidal symptoms). One patient stopped the treatment without prior medical advice.

## 4. Discussion

In this study, we performed the first retrospective chart review of lurasidone augmentation of clozapine treatment effects in a group of treatment-resistant SZ patients. We have provided a detailed clinical description of 16 SZ individuals treated with the use of this combination for a period from 2 to 38 months. Our results showed a significant reduction in overall illness severity after the first and second months of this treatment. The vast majority of patients (14/16, 87.5%) responded to lurasidone augmentation of clozapine, presenting an observable reduction in positive, depressive and anxiety symptoms, as well as improvements in psychosocial functioning.

The results of our retrospective chart review show that the overwhelming majority of patients (91 out of 112 (81.25%)) using clozapine required its augmentation with another antipsychotic. So far, the largest number of published studies evaluated the use of clozapine in combination with risperidone, aripiprazole and amisulpride; however, the majority of the analysis concluded that augmentation of clozapine with a second antipsychotic has a modest or little clinical effect [10,29,30,31].

Even though lurasidone gained approval for the treatment of schizophrenia in adult patients in the United States in 2010 [32], there is only one study evaluating the effects of lurasidone augmentation of clozapine in this clinical group. Arienti et al. (2021) presented a case report of two SZ patients treated with the use of this combination [16]. The authors used PANSS to assess general psychopathology, both at the baseline and at discharge. The first case presented was a 44-year-old male, with the onset of SZ symptoms at the age of 22. The patient presented persistent negative symptoms, such as autistic withdrawal, abulia, flattened affect and anhedonia with significant impairment in psychosocial functioning. There were four ineffective antipsychotic treatment trials in the past (haloperidol, quetiapine, clotiapine and paliperidone). During the exacerbation of positive symptoms and an episode of psychomotor agitation, clozapine monotherapy was introduced at a daily dose of 500 mg. During the next recurrence of positive symptoms, clozapine was augmented with lurasidone titrated up to 74mg daily. The treatment resulted in a significant reduction in those symptoms, with a partial clinical response. During another psychotic episode, the patient’s positive and negative symptoms gradually decreased when the dose was titrated up to 148 mg daily. The second case presented was a 54-year-old male with a 34-year course of SZ. There were four ineffective antipsychotic trials in the past (olanzapine, risperidone and quetiapine). Six years after a good clinical response to clozapine, which was used at doses up to 300 mg daily, positive symptoms recurred. Augmentation of clozapine with lurasidone titrated up to 74 mg resulted in a reduction in symptoms in the positive, negative and affective domains and a decrease in cognitive impairment after 7 days of treatment. None of the abovementioned patients presented marked side effects associated with the use of that combination.

Our study adds further evidence for the beneficial effects of lurasidone augmentation of clozapine in a group of 16 SZ patients. The cases described in our retrospective chart review presented treatment resistance to a median of five previous ineffective trials. Moreover, contrary to the work of Arienti et al. in 2021, patients were also resistant to common strategies for augmentation of clozapine [11,16] with the use of different antipsychotic drugs: amisulpride (five patients), aripiprazole (four patients), haloperidol (one patient) and risperidone (one patient). Out of 16 patients, 14 (87.5%) responded to the add-on of lurasidone (daily dose range 37–148 mg, median 104,1 mg) for augmentation of clozapine (daily dose range 50–500 mg, median 326.56 mg) treatment after a median of 6 weeks. Significant improvement in the global illness severity reflected in CGI-S scores was observable after one month of treatment, with further amelioration in the second month. As in the cases presented by Arienti et al. in 2021, a significant reduction in positive symptoms was noticed during pharmacotherapy with the use of this combination in our study [16]. This improvement was observed in the case of 10 patients. Moreover, an observable decrease in the severity of anxiety (seven patients) and affective symptoms (seven patients), as well as sexual dysfunctions (two patients), was described. Although the analyzed medical records did not directly describe a reduction in negative symptoms, a noticeable improvement in psychosocial functioning was observed in the case of nine individuals; e.g., patients were more willing to participate in ward activities and more eager to make social contacts, and four of them went back to work.

Lurasidone augmentation of clozapine was relatively well tolerated among the analyzed cases. Two out of sixteen patients were required to discontinue the treatment due to side effects. The first of them developed hyperprolactinemia with galactorrhea, and the second one revealed significant extrapyramidal symptoms. No hematological side effects were observed in blood tests performed during the period of observation. Additionally, it is noteworthy that in one case, the add-on of lurasidone resulted in the possibility of reducing the daily clozapine dose from 300 to 225 mg. During the treatment, this patient presented the normalization of prolactin, glucose and HbA1 levels and achieved a weight reduction from 145 to 134 kg during 5 weeks of treatment. Further studies are required to evaluate the side effects, as well as positive metabolic effects, of lurasidone augmentation of clozapine treatment.

Both a partial explanation of our positive preliminary results and the justification for further studies may be the significant pharmacodynamic complementarity of clozapine and lurasidone, as well as the high level of safety of lurasidone, making their combination a rational pharmacotherapeutic option. Both medications act as antagonists of D_2_, 5HT_7_ and α_2_ receptors, but lurasidone’s affinity is much higher. Lurasidone’s affinity for 5HT_1A_ is also significantly higher than clozapine’s. On the other hand, contrary to clozapine, lurasidone has negligible affinity for histamine H_1_, dopamine D_1_, serotonin 5HT_2C_, adrenergic α_1_ and different subtypes of muscarinic receptors [33,34,35].

We are aware of the limitations of our study: (a) a relatively small number of cases, (b) heterogeneity of the studied population, e.g., variability in the treatment duration, (c) different doses of clozapine used and no access to clozapine blood levels for most of the patients. Apart from CGI-S and CGI-I, we did not use specific clinical tools to evaluate positive, negative, anxiety and affective symptoms or measures of psychosocial functioning. Data about the improvement in the abovementioned areas were derived from patients’ medical records. Due to the abovementioned limitations, no strong conclusions can be drawn. However, due to the significant research gap on this subject, we believe our study provides valuable information that can be used in future randomized controlled trials evaluating the effects of lurasidone augmentation of clozapine.

## 5. Conclusions

Our retrospective chart review provides the largest up-to-date evaluation of lurasidone augmentation of clozapine in patients with treatment-resistant SZ in a “real-world” setting. The vast majority of patients responded to the add-on of lurasidone and did not present noticeable side effects. The use of the abovementioned combination was associated with a reduction in positive, depressive and anxiety symptoms, as well as improvements in psychosocial functioning. There is a strong need to create well-designed clinical studies evaluating the efficacy of new strategies for clozapine augmentation in treatment-resistant SZ. Future studies are required to verify the usability of the lurasidone and clozapine combination in the treatment of this disorder.

## Figures and Tables

**Table 1:** Detailed description of schizophrenia patients treated with lurasidone augmentation of clozapine. The table has been attached as a separate file to make it easier to view, Table S1.

Case numbers	1	2	3	4	5	6	7	8	9	10	11	12	13	14	15	16
Age	27	42	42	35	37	45	40	38	36	42	28	29	23	40	27	38
Sex	M	M	F	F	M	F	M	F	F	F	M	M	M	M	M	F
Duration of illness	4	22	13	12	18	16	16	10	14	20	7	3	7	16	1	12
Number of previous ineffective pharmacotherapy trials prior to the use of clozapine +lurasidone combination	4	3	5	6	3	2	7	5	4	6	7	7	2	5	2	8
Dose of clozapine that was combined with lurasidone	500 mg	450 mg	325 mg	300 mg	300 mg	500mg	100 mg	50 mg	400 mg	425 mg	300 mg	500 mg	375 mg	100 mg	400 mg	200 mg
Antipsychotic used in combination with clozapine prior to switch to lurasidone	Initially lurasidone + olanzapine, subsequent gradual switch from olanzapine to clozapine	Amisulpride 300 mg	Amisulpride 600 mg	Haloperidol 3 mg	Risperidone 4 mg	Amisulpride 400 mg	Amisulpride 600 mg	Aripiprazole 15 mg	Aripiprazole 30 mg	Cariprazine 4,5 mg	Aripiprazole 30 mg	Amisulpride	Aripiprazole + olanzapine prior to switch to clozapine +lurasidone	Amisulpride + lurasidone (with subsequent switch from amisulpride to clozapine)	Olanzapine + lurasidone (with subsequent switch from olanzapine to clozapine)	Aripiprazole
Somatic comorbidities	-	-	-	Hypothyroidism	Obesity, hypercholesterolemia, hypertension, impaired glucose tolerance		-	-	Diabetes	-	-	-	-	Chronic myeloid leukemia, hypercholesterolemia, obesity	-	Hyperprolactinemia
Addictions (ICD-10 code)	F17	F10 In remission	-	-	F19 In remission	-	-	-	-	F17	-	F17, F12 in remission	-	-	-	-
Other psychotropic medications used at the time of adding the antipsychotic (reason for their use)	-	Pregabalin 600 mg, vortioxetine 10mg	Lamotrigine 150 mg, vortioxetine 10mg Opipramol 150 mg	Lamotrigine 100 mg	Trazodone 100 mg	-	-	Vortioxetine 10 mg, lamotrigine 200 mg/d	-	Pregabalin 300 mg, lorazepam 2 mg, Biperiden 4 mg	Biperiden 4 mg	Biperiden 6mg (due to extrapyramidal symptoms during antipsychotic treatment), Lamotrigine 100 mg	-	-	-	Pregabalin 150 mg, sertraline 100 mg (anxiolytic treatment)
Nonpsychiatric medications (with daily doses)	Bisoprolol 5 mg	-	-	Levothyroxine 125 ug	Rosuvastatin 40 mg, clofibrate fenofibrate 215 mg, nebivolol 5 mg, metformin 1000 mg	Lamotrigine 175 mg			Metformin 1000 mg	Bisoprolol 5 mg	-	Propranolol 40 mg/d	-	Nilotinib 800 mg/d	-	Propranolol 30 mg/d
Initial dose of lurasidone	37 mg	37 mg	37 mg	37 mg	37 mg	37 mg	37 mg	37 mg	37 mg	37 mg	37 mg	37 mg	37 mg	37 mg	37 mg	37 mg
Final dose of lurasidone	148 mg	148 mg	148 mg	37 mg	74 mg	74 mg	148 mg	37 mg	74 mg	74 mg	111 mg	74 mg	148 mg	148 mg	111 mg	111 mg
Duration of the combined treatment (clozapine + lurasidone), in months	14	14	38	8	12	18	6	4	4	2	2	4	9	17	3	12
	7	5	6	5	5	7	6	5	6	6	7	6	6	6	7	6
	5	2	3	1	4	5	3	4	5	5	5	5	5	4	5	4
	2	1	1	2	3	2	1	1	2	2	2	3	2	2	2	2
Number of weeks until the observable therapeutic effect was achieved	4	5	3	4	6	12	8	6	8	4	6	6	8	6	4	4
Effects of the addition of lurasidone	Reduction in positive symptoms, remission of anxiety, mood improvement; the patient was more eager to make social contacts and decided to start treatment in a day care unit.	Remission of sexual dysfunctions (decreased libido and erectile dysfunctions); Remission of depressive and anxiety symptoms; Reduction in intensity of ideas of reference; Improved level of functioning. The patient returned to work activity.	Further gradual improvement throughout the duration of treatment; Returned to work.	Complete remission after 2 months of the combined treatment (in all symptom domains); Marked reduction in positive symptoms, greater emotional stability, decreased level of anxiety and excessive worrying, improved level of activity; Stabilization of weight; Overall improvement in functioning and social interactions.	Normalization of prolactin, glucose and HbA1 levels; Possibility of clozapine daily dose reduction from 300 to 225 mg; Weight reduction (from 145 to 134 kg for 5 weeks); After 10 months, recurrence of depressive symptoms—treated with 60 mg of duloxetine, without worsening of positive symptoms; Partial improvement in sexual functions; Weight reduction (from 144 to 121 kg).	Improved: emotional reactivity, psychomotor drive, motivation to pursue activities, spontaneity; Overall improvement in functioning and social interaction. Normalization of the prolactin level.	Significant improvement, less anxiety; the patient returned to work.	Significant mood improvement.	Improvement in mood, level of activity, intensity of hallucinations.	Improvement in affective symptoms, better modulation of affect, significant reduction in positive symptoms (delusions and hallucinations), improved level of functioning: the patient was planning to return to work, was more eager to make social contacts.	Significant reduction in positive symptoms and anxiety. The patient decided to start treatment in a day care unit.	Moderate improvement in positive symptoms and level of activity (the patient was more willing to participate in ward activities, made contacts with other patients). Due to extrapyramidal symptoms (tremor in upper limbs), lurasidone was discontinued.	Improvement in mood, reduction in positive symptoms, decreased level of anxiety, more social contacts.	Reduction in frequency and intensity of positive symptoms, improved level of social and occupational functioning	Improvement in mood, reduction in positive symptoms and anxiety, remission of suicidal thoughts and behaviors; After 3 months of treatment, the patient discontinued the medications without consulting with his doctor (the patient presented little insight into the disease and his symptoms).	Significant reduction in positive symptoms and anxiety level, improved level of functioning; Due to symptomatic hyperprolactinemia (with galactorrhea), lurasidone was discontinued and switched to aripiprazole.

**Table 2:** Summarized description of the schizophrenia patient group treated with lurasidone augmentation of clozapine.

	Treatment-Resistant SZ Patients Undergoing Lurasidone Augmentation of Clozapine Treatment (n = 16)
Age (median number of years, (25th percentile–75th percentile))	37.5 (28.25–41.5)
Sex (women/men)	7/9
Duration of SZ treatment (median number of weeks, (25th percentile–75th percentile))	12.5 (7–16)
Number of previous unsuccessful pharmacotherapies before introducing lurasidone augmentation of clozapine	5 (3–6.75)
Initial lurasidone dose (mean mg, (SD))	37 (0)
Final lurasidone dose (mean mg, (SD))	104.1 (41)
Clozapine dose (mean mg, (SD))	326.6 (147,3)
CGI-S baseline (mean (SD))	6 (0.73)
CGI-S final (mean (SD))	4.19 (1.33)
CGI-I (mean (SD))	1.88 (0.62)
Duration of lurasidone augmentation of clozapine treatment (median number of weeks, (25th percentile–75th percentile))	8.5 (4–14)
Number of weeks of treatment until observable therapeutic effect (median, (25th percentile–75th percentile))	6 (4–6)
Number of patients responding to treatment (n, (%))	14 (87.5%)
Discontinuation of treatment due to the side effects (n, (%))	2 (12.5%)
Discontinuation without prior medical advice (n, (%))	1 (6.25%)

**Table 3:** Characteristics of the psychopathological symptoms and somatic conditions presented by schizophrenia patients before initiation of lurasidone augmentation of clozapine.

Case Number	1	2	3	4	5	6	7	8	9	10	11	12	13	14	15	16
Residual positive symptoms	X	X	X	X		X	X		X	X		X	X	X		X
Exacerbation of positive symptoms		X							X	X	X				X	
Negative symptoms	X		X	X		X				X	X					
Depressive symptoms		X	X	X	X		X	X					X		X	
Anxiety	X	X	X				X			X			X		X	X
Suicidal thoughts	X														X	
Cognitive dysfunctions			X	X								X				
Sexual dysfunctions		X	X		X											
Hyperprolactinemia			X		X	X										X
Increased appetite and body weight/obesity				X	X	X								X		
Disorders of glucose metabolism				X	X											

## Data Availability

Data are unavailable due to privacy or ethical restrictions.

## Supplementary Materials

The following supporting information can be downloaded at: https://www.mdpi.com/article/10.3390/brainsci13030445/s1. Table S1: Detailed description of schizophrenia patients treated with lurasidone augmentation of clozapine. Annex1: Data-collecting forms.
