# Supplementary material for: Lurasidone Augmentation of Clozapine in Schizophrenia—Retrospective Chart Review
Source: Brain Sci. 2023 Mar 4;13(3):445. doi: 10.3390/brainsci13030445 (PMC10046327; doi:10.3390/brainsci13030445)
Supplement: Supplementary file 1 [file brainsci-13-00445-s001.zip › Annex 1.pdf]

Annex 1

Data collecting form for all patients on clozapine:

Patient code	Age	Gender	Diagnosis	Clozapine dose (mg)	Treatment schedule (1 monotherapy; 2-politherapy)	Other antipsychotics with dosing

Form for patients on clozapine and lurasidone combination:

Case number	
Illness duration (in years)	
Number of previous ineffective pharmacotherapy trials prior to the use of clozapine +lurasidone combination	
Antipsychotic used in combination with clozapine prior to switch to lurasidone	
Somatic comorbidities	
Addictions	
Other psychotropic medications used at the time of adding the antipsychotic	
Nonpsychiatric medications (with daily doses)	
Initial dose of lurasidone	
Target dose of lurasidone	
Duration of the combined treatment (clozapine + lurasidone), in months	
Clozapine dose (mg)	
**Symptoms at the beginning of the combined clozapine + lurasidone treatment:**	
Residual positive symptoms (1-yes; 0-no)	
Exacerbation of positive symptoms (1-yes; 0-no)	
Negative symptoms (1-yes; 0-no)	
Depressive symptoms (1-yes; 0-no)	
Anxiety (1-yes; 0-no)	
Suicidal thoughts (1-yes; 0-no)	
Cognitive dysfunctions (1-yes; 0-no)	
Sexual dysfunctions (1-yes; 0-no)	
Hyperprolactinemia (1-yes; 0-no)	
Overweight/Obesity (1-yes; 0-no)	
Disorders of glucose metabolism (1-yes; 0-no)	
