# Supplementary material for: Lurasidone Augmentation of Clozapine in Schizophrenia—Retrospective Chart Review
Source: Brain Sci. 2023 Mar 4;13(3):445. doi: 10.3390/brainsci13030445 (PMC10046327; doi:10.3390/brainsci13030445)
Supplement: Supplementary file 1 [file brainsci-13-00445-s001.zip › Table S1.pdf]

Table S1

Case numbers	1	2	3	4	5	6	7	8	9	10	11	12	13	14	15	16
**Age**	27	42	42	35	37	45	40	38	36	42	28	29	23	40	27	38
**Sex**	M	M	F	F	M	F	M	F	F	F	M	M	M	M	M	F
**Duration of illness**	4	22	13	12	18	16	16	10	14	20	7	3	7	16	1	12
**Number of previous ineffective pharmacotherapy trials prior to the use of clozapine +lurasidone combination**	4	3	5	6	3	2	7	5	4	6	7	7	2	5	2	8
**Dose of clozapine that was combined with lurasidone**	500mg	450mg	325mg	300mg	300mg	500mg	100 mg	50 mg	400mg	425 mg	300 mg	500 mg	375 mg	100 mg	400 mg	200 mg
**Antipsychotic used in combination with clozapine prior to switch to lurasidone**	Initially lurasidone + olanzapine, subsequent gradual switch from olanzapine to clozapine	amisulpride 300mg	amisulpride 600mg	haloperidol 3mg	risperidone 4mg	amisulpride 400mg	amisulpride 600 mg	aripiprazole 15 mg	aripiprazole 30 mg	cariprazine 4,5 mg	aripiprazole 30 mg	amisulpride	aripiprazole + olanzapine prior to switch to clozapine +lurasidone	amisulpride + lurasidone (with subsequent switch from amilsupride to clozapine)	olanzapine + lurasidone (with subsequent switch from olanzapine to clozapine)	aripiprazole
**Somatic comorbidities**	-	-	-	Hypothyroidism	Obesity, hypercholesterolaemia, hypertension, impaired		-	-	Diabetes	-	-	-	-	Chronic myeloid leukemia, hypercholesterolemia, obesity	-	hyperprolactinemia

					glucose tolerance											
**Addictions (ICD-10 code)**	F17	F10<br>In remission	-	-	F19<br>In remission	-	-	-	-	F17	-	F17, F12 in remission	-	-	-	-
**Other psychotropic medications used at the time of adding the antipsychotic**<br>**(reason for their use)**	-	pregabalin 600mg<br>vortioxetine 10mg	lamotrigine 150mg*<br>vortioxetine 10mg<br>Opipramol 150mg	lamotrigine 100mg	trazodone 100mg	-	-	vortioxetine 10 mg<br>lamotrigine 200 mg/d	-	pregabalin 300 mg, lorazepam 2 mg,<br>Biperiden 4 mg	biperiden 4 mg	biperiden 6mg (due to extrapyramidal symptoms during antipsychotic treatment) Lamotrigine 100 mg	-	-	-	pregabalin 150 mg, sertraline 100 mg (anxiolytic treatment)
**Nonpsychiatric medications (with daily doses)**	bisoprolol 5 mg	-	-	levothyroxine 125ug	rosuvastatin 40mg clofibrate fenofibrate 215 mg nebivolol 5 mg, metformin 1000mg	lamotrigine 175mg*			metformin 1000mg	bisoprolol 5 mg	-	propranolol 40 mg/d	-	nilotinib 800 mg/d	-	propranolol 30 mg/d
**Initial dose of lurasidone**	37mg	37mg	37mg	37mg	37mg	37mg	37mg	37mg	37mg	37 mg	37 mg	37 mg	37 mg	37 mg	37 mg	37 mg
**Final dose of lurasidone**	148 mg	148mg	148mg	37mg	74mg	74mg	148 mg	37 mg	74 mg	74 mg	111 mg	74 mg	148 mg	148 mg	111 mg	111 mg
**Duration of the combined treatment (clozapine + lurasidone), in months**	14	14	38	8	12	18	6	4	4	2	2	4	9	17	3	12
	7	5	6	5	5	7	6	5	6	6	7	6	6	6	7	6

	5	2	3	1	4	5	3	4	5	5	5	5	5	4	5	4
	2	1	1	2	3	2	1	1	2	2	2	3	2	2	2	2
**Number of weeks until the observable therapeutic effect was achieved**	4	5	3	4	6	12	8	6	8	4	6	6	8	6	4	4
**Effects of the addition of lurasidone**	Reduction of positive symptoms, remission of anxiety, mood improvement, the patient was more eager to make social contacts and decided to start treatment in a day care unit.	Remission of sexual dysfunctions (decreased libido and erectile dysfunctions)<br>Remission of depressive and anxiety symptoms<br>Reduction in intensity of ideas of reference<br>Improved level of functioning.<br>The patient return to work activity.	further gradual improvement throughout the duration of treatment<br>Return to work	Complete remission after 2 months of the combined treatment (in all symptom domains)<br>Marked reduction of positive symptoms, greater emotional stability, decreased level of anxiety and excessive worrying, improved level of activity<br>Stabilisation of weight<br>Overall improvement in functioning and social interactions	Normalisation of prolactin, glucose and HbA1 levels<br>Possibility of clozapine daily dose reduction from 300 to 225 mg<br>Weight reduction (from 145 to 134 kg for 5 weeks)<br>After 10 months recurrence of depressive symptoms – treated with 60 mg of duloxetine, without worsening of positive symptoms<br>Partial improvement	Improved: emotional reactivity, psychomotor drive, motivation to pursue activities, spontaneity<br>Overall improvement in functioning and social interaction<br>Normalisation of the prolactin level.	Significant improvement, less anxiety, the patient return to work	Significant mood improvement	Improvement in mood, level of activity, intensity of hallucinations	Improvement in affective symptoms, better modulation of affect, significant reduction of positive symptoms (delusions and hallucinations) , improved level of functioning – the patient was planning to return to work, ws more eager to make social contacts	Significant reduction of positive symptoms and anxiety. The patient decided to start treatment in a day care unit	Moderate improvement of positive symptoms and level of activity (the patient was more willing to participate in ward activities, made contacts with other patients).<br>Due to extrapyramidal symptoms (tremor in upper limbs) lurasidone was discontinue	Improvement in mood, reduction of positive symptoms, decreased level of anxiety, more social contacts	Reduction of frequency and intensity of positive symptoms, improved level of social and occupational functioning	Improvement in mood, reduction of positive symptoms and anxiety, remission of suicidal thoughts and behaviours<br>After 3 months of treatment the patient discontinued the medications, without consulting it with his doctor (the patient presented little insight in the disease and his symptoms).	Significant reduction of positive symptoms and anxiety level, improved level of functioning<br>Due to the symptomatic hyperprolactinemia (with galactorrhoea)' lurasidone was discontinued and switched to aripiprazole.

	of sexual functions<br><br>Weight reduction (ftom 144 to 121 kg)	
